# Outbreak of Leptospirosis in Kerala

**DOI:** 10.3126/nje.v8i4.23876

**Published:** 2018-12-31

**Authors:** Sruthi James, Brijesh Sathian, Edwin van Teijlingen, Mohammad Asim

**Affiliations:** 1 Research Coordinator, Clinical Research, Trauma Surgery, Surgery, Hamad General Hospital, Doha, Qatar.; 2 Academic Research Associate, Clinical Research, Trauma and Vascular Surgery, Surgery Department, Hamad General Hospital, Doha, Qatar.; 3 Professor, Centre for Midwifery, Maternal and Perinatal Health, Bournemouth University, Bournemouth, UK.

In South Asia, the monsoon brings life to vegetation, but at the same time has potential to cause public health problems. Notably, the climate change due to global warming is affecting the extent of monsoon rainfall in the region causing flooding which increases the risks of major disease outbreaks. Flooding and standing water after heavy rainfall increases the risk of vector-borne diseases such as dengue, malaria, plague, chikungunya, typhoid, cholera and Leptospirosis.

Worldwide, Leptospirosis is one of the most common and emerging zoonoses, except on the North and South Poles. Rat fever or leptospirosis is a bacterial infection caused by the spiral-shaped bacteria (spirochete) of the genus *Leptospira* [1]. This infection is mainly seen in wild and even domesticated species of rodents. It is mainly transmitted to humans by exposure of the mucous membranes (oral, nasal & eye) and skin abrasions or cuts to the urine or tissues of infected rodents or soil contaminated by their urine [2]. Rats are the primary reservoir of leptospirosis, although farm animals and livestock, such as horses, pigs, dogs or cattle, and even wild animals can also be a reservoir for the bacteria. However, human-to-human transmission seems to occur occasionally [1]. It is also an occupational hazard with potential risk of exposure among outdoors workers such as farmers, cleaners, veterinarians, agricultural workers. Moreover, there exists an increased chance of a recreational hazard to those who swims and wades in contaminated waters [1, 2].

The clinical presentation of mild leptospirosis usually manifested with various symptoms such as fever, chills, headache, muscle aches, vomiting, or diarrhea or some cases remain asymptomatic. Many of these clinical symptoms are non-specific and may leads to consideration of other possible diagnoses. Therefore, serologic testing should be done in acute and convalescent phase to confirm the diagnosis. Of note, there are chances of relapse (fulminant) which is manifested with severe life-threatening illness resulted in meningitis, hepatic or renal failure and pulmonary hemorrhage [2]. The average incubation period from exposure to symptomatic presentation ranges from 7 to 12 days with a maximum range of 20-30 days [1].

The mild disease is self-limiting which resolve spontaneously with time. Whilst the management of severe leptospirosis necessitates early administration of antimicrobial therapy using doxycycline or penicillin, to control disease progression. However, like most public health problems, the primary prevention depends on the avoidance of leptospirosis exposure through awareness of transmission mechanisms. Precautions should be taken to prevent the bacterial invasion by using protective clothes and foot wears among occupational workers at high-risk of exposure to the contaminated water or engaging in recreational activities [3].

In South Asia, it is often spread after heavy rain or post flooding. A systematic review, estimated higher rate of morbidity and mortality due to leptospirosis in the South and Southeast Asian regions, as it is an under-reported public health concern [4]. Earlier studies from India have shown that the incidence and consequences of leptospirosis is increasing, however studies on prognosis of the disease are still fairly rare [5,6]. It was declared as a public health problem in India from 1980 onwards, and isolated cases have been reported prior to this date [7]. Several outbreaks of Leptospirosis have been reported from Tamil Nadu, Gujarat, and Karnataka. Other states of India have reported the incidence of sporadic leptospirosis cases.

Rat fever has long been a major threat to the State of Kerala with more than 1,000 cases is being reported annually. Nationally, it causes the highest number of deaths among all communicable diseases in the state of Kerala. At least 100 deaths were reported yearly in Kerala before 2010. In 2006, there were 1,821 cases of rat fever of which 104 (5.7%) died and in 2007 there were 1,359 cases with 229 (16.9%) deaths. The number of leptospirosis cases in 2008, 2009 and 2010 were 1305, 1237 and 1016 with mortality rate of 136 (10.4%), 107 (8.6%) and 85 (8.4%), respectively [8]. In 2011 and 2012, the number of confirmed cases was 944 and 736 with death rate of 70 (7.4%) and 18 (2.4%). It has been also reported that in 2013 and 2014, confirmed cases was 814 and 717 with 34 (4.2%) and 19 (2.6%) deaths respectively [9]. Notably, the incidence and mortality of leptospirosis in Kerala for the following years showed a declining trend as compared to the previous years. In 2015, 43 people died of rat fever and in the subsequent years the death toll was found to be 35 and 80 in 2016 and 2017, respectively. From the month of January till July 2018 (before the flood), 28 deaths were reported due to leptospirosis in Kerala.

Kerala suffered unusually heavy rainfall and faced a catastrophic flood in August and September 2018, in which around 500 people died. The aftermath of the flood brings several epidemics. The Directorate of Health Services delivered an action plan for the prevention and control of communicable diseases and informed the public regarding the symptoms and appropriate treatment of such diseases with the help of volunteers. Despite that, there was a major threat of outbreak of leptospirosis in Kerala after this floods and the highest number of leptospirosis cases was reported from Kozhikode district of Kerala which was affected most by the flood. It was reported that leptospirosis, and dengue fever has killed more than 70 people in shorter time span.

As of September 11, 2018, the Integrated Disease Surveillance Project (IDSP) data revealed that there were 2598 suspected leptospirosis cases with 95 suspected deaths, whereas the confirmed cases stood at 1318 with a confirmed death rate of 53 (4.0%). On the other hand, statistics from the Kerala State Health Department reported 570 confirmed cases and 18 (3.2%) confirmed deaths; however the suspected cases were 1107 with 33 suspected deaths since 1 September 2018 [7].

Due to global warming and deforestation, heavy rainfall and flooding is more likely to be seen in the South Asia region in general and Kerala in particular. It is therefore important to promote the public health awareness for primary prevention with proper sanitation, hand washing, wearing protective clothing, drinking boiled water, and avoid contact with contaminated water or soil, which are the common sources of exposure. Moreover, precautions should be taken to prevent the bacterial invasion by using protective clothes and foot wears among occupational workers at high-risk of exposure to the contaminated water.
